# Effects of Age and Sex on Optic Nerve Sheath Diameter in Healthy Volunteers and Patients With Traumatic Brain Injury

**DOI:** 10.3389/fneur.2020.00764

**Published:** 2020-08-07

**Authors:** Danilo Cardim, Marek Czosnyka, Karthikka Chandrapatham, Rafael Badenes, Alessandro Bertuccio, Anna Di Noto, Joseph Donnelly, Paolo Pelosi, Lorenzo Ball, Peter J. Hutchinson, Chiara Robba

**Affiliations:** ^1^Brain Physics Laboratory, Division of Neurosurgery, Department of Clinical Neurosciences, Addenbrooke's Hospital, University of Cambridge, Cambridge, United Kingdom; ^2^Institute for Exercise and Environmental Medicine, Texas Health Presbyterian Hospital Dallas, Dallas, TX, United States; ^3^Department of Neurology and Neurotherapeutics, University of Texas Southwestern Medical Center, Dallas, TX, United States; ^4^Institute of Electronic Systems, Warsaw University of Technology, Warsaw, Poland; ^5^Department of Surgical Sciences and Integrated Diagnostics, University of Genoa, Genoa, Italy; ^6^University of Valencia Hospital Clinic, Anesthesiology and Surgical-Trauma Intensive Care, Valencia, Spain; ^7^Department of Neurosurgery, S. Cesare, Arrigo, Antonio, Biagio, Alessandria, Italy; ^8^Anaesthesia and Intensive Care, San Martino Policlinico Hospital, IRCCS for Oncology and Neurosciences, University of Genoa, Genoa, Italy; ^9^Department of Anesthesiology, University of Auckland, Auckland, New Zealand; ^10^Department of Surgical Sciences and Integrated Diagnostics, University of Genoa, Genoa, Italy; ^11^Division of Neurosurgery, Department of Clinical Neurosciences, Addenbrooke's Hospital, University of Cambridge, Cambridge, United Kingdom

**Keywords:** optic nerve sheath diameter, ultrasonography, traumatic brain injury, intracranial pressure, healthy volunteers

## Abstract

The measurement of optic nerve sheath diameter (ONSD) has been reported as a non-invasive marker for intracranial pressure (ICP). Nevertheless, it is uncertain whether possible ONSD differences occur with age and sex in healthy and brain-injured populations. The aim of this study was to investigate the effects of sex and age on ONSD in healthy volunteers and patients with traumatic brain injury. We prospectively included 122 healthy adult volunteers (Galliera Hospital, Genova, Italy), and compared age/sex dependence of ONSD to 95 adult patients (Addenbrooke's Hospital, Cambridge, UK) with severe traumatic brain injury (TBI) requiring intubation and invasive ICP monitoring. The two groups were stratified for sex and age. Age was divided into 3 subgroups: (1) young adults: 18–44 years; (2) middle-aged adults: 45–64 years; (3) old adults: >65 years. In healthy volunteers, ONSD was significantly different between males and females [median (interquartile range): 4.2 (3.9–4.6) mm vs. 4.1 (3.6–4.2) mm (*p* = 0.01), respectively] and was correlated with age (R = 0.50, *p* < 0.0001). ONSD was significantly increased in group 3 compared to groups 2 and 1, indicating that ONSD values are higher in elderly subjects. In TBI patients, no differences in ONSD were found for sex and the correlation between ONSD and age was non-significant (R = 0.13, *p* = 0.20). ONSD increases with age and is significantly larger for males in healthy volunteers but not in TBI patients. Different ONSD cut-off values need not be age- or sex-adjusted for the assessment of increased ICP in TBI patients.

## Introduction

Elevated intracranial pressure (ICP) is a harmful condition resulting from many neurological and non-neurological diseases (1) and is associated with poor outcome (2). The gold standard techniques to measure intracranial pressure (ICP) are all invasive, such as intraventricular catheterization and intraparenchymal probes. They carry several risks including infection, hemorrhage and therefore, are precluded in patients in need of monitoring over long time or susceptible to coagulopathy or platelet disorders (3).

In this scenario, several alternative non-invasive methods to assess ICP have been developed over the past years (4). Among these, the ultrasonographic measurement of optic nerve sheath diameter (ONSD) has gained popularity given its feasibility, repeatability, safety and absence of radiation hazard or known side effects. The sheath around the optic nerve is an extension of the dura-mater and is filled with cerebrospinal fluid (CSF), so increases of intracranial pressure are detectable as rises of ONSD in the anterior, retrobulbar compartment ~3 mm behind the globe (5). However, there are potential challenges: the extension of optic sheath is most probably a non-linear function of ICP, its elasticity is unknown and may vary from person-to-person and many variables may affect the relationship between diameter and ICP.

This technique has demonstrated low intra- and inter-observer variability (6) and according to several studies, ICP and ONSD are linearly associated in conditions of intracranial hypertension (4, 7–9). A recent meta-analysis (10) indicated that ultrasonographic ONSD may be a potentially useful approach to assess intracranial hypertension when invasive devices are not indicated or available. However, the threshold of ONSD related to elevated ICP in brain-injured patients is still debatable (10). More information is needed about the variability of ONSD in pathological conditions and the range of normality. Furthermore, it is not clear whether non-invasive ICP assessment with ONSD in TBI patients is affected by age and sex.

The aim of this study was to investigate the effects of sex and age on optic nerve sheath diameter in healthy volunteers and patients with traumatic brain injury.

## Materials and Methods

This article fulfills the “Strengthening the reporting of observational studies in epidemiology (STROBE)” guidelines (11).

### Healthy Population

The study protocol was approved by the Research Ethics Boards of Genova, Italy (REC 031R8G2015) and observed the principles of the Declaration of Helsinki. One-hundred twenty-two healthy volunteers were prospectively recruited between 1st September 2015 and 1st January 2018 at Galliera Hospital, Genova, Italy. We prospectively recruited adult (>18 years-old) Italian healthy volunteers [American Society of Anesthesiologists (ASA) 1–2] undergoing pre-surgery examination for low risk surgical interventions at Galliera Hospital. Inclusion criteria included the absence of any cardiovascular, respiratory neurological and systemic pathology and the absence of any chronic diseases and any acute illnesses in the preceding 4 weeks from the assessment. Written informed consent was obtained from all participants before enrollment. Patients with a history of head injury, history of optic nerve lesion or previous optic nerve trauma, as well as pregnant women (excluded by anamneses and/or laboratory test) were excluded. Eligible patients were screened, their demographic data recorded, as well as non-invasive arterial blood pressure (ABP) measured using a conventional arm cuff.

### TBI Patients

Ninety-five TBI patients were recruited between the 20th December 2015 and the 1st September 2017 at the Neurosciences and Trauma Critical Care Unit, Addenbrooke's Hospital, Cambridge, UK. The protocol was approved by the Research Ethics Boards at the University of Cambridge (REC 15/lo/1918). Written consent was obtained from all participants' next of kin. We included patients aged >18 years old with severe traumatic brain injury [Glasgow Coma Scale (GCS) 3–8], requiring intubation and invasive ICP monitoring.

Exclusion criteria were the absence of an informed consent, skull base fracture with cerebrospinal fluid leaking and a known history of ocular pathology or optic nerve trauma. Demographic data including sex, age, ABP measured invasively at the radial artery (Baxter Healthcare Health Care Corp., Cardio Vascular Group, Irvine, CA, USA), ICP [via an intraparenchymal probe (Codman & Shurtleff, Raynham, MS, USA)] and ONSD were recorded after the ICP probe insertion on admission day 1 at variable times across individuals according to the patients logistic availability.

### Endpoints

The primary endpoint was to evaluate ONSD measured by ultrasound in healthy volunteers. We aimed to assess the difference in ONSD between sex (male vs. female) and in different age groups [group 1: young adults (18–44 years); group 2: middle-aged adults (45–64 years); group 3: old adults (>65 years)]. The secondary endpoint was to assess the differences in ONSD stratified for age and sex patients with traumatic brain injury and compare these findings with the healthy volunteers population.

### ONSD Measurement

In healthy volunteers and TBI patients, ONSD was measured 3 mm behind the retina. Two trained investigators with more than 30 examinations of experience as previously described (5) (CR, FC in Genova and CR, DC in Cambridge) used a 7.5 MHz linear ultrasound probe oriented perpendicularly in the vertical plane and at around 30 degrees in the horizontal plane on the closed eyelids of both eyes from supine subjects (Cambridge, UK: 11L4, Xario 200; Toshiba, Zoetermeer, The Netherlands; Genova Italy: DC-T6, Mindray Medica, Schenzen, China). Ultrasound gel was applied on each eyelid and recordings were performed in the axial and longitudinal planes. An electronic caliper was used to mark 3 mm perpendicularly behind the retina and the ONSD was measured at the depth marker at right angles to the optic nerve. The final ONSD measurement was calculated as the average of the transversal and sagittal diameters for both eyes.

### Statistical Analysis

Statistical analyses were performed using RStudio software (version 3.6.0). Continuous variables were expressed as median [interquartile range (IQR)]. Data were checked for normality using the Shapiro-Wilk test. Pearson or non-parametric Spearman correlation coefficients were used to relate continuous variables. Welch Two Sample *t*-test or non-parametric Wilcoxon rank sum test were applied to evaluate the differences in ONSD concerning sex in healthy volunteers and TBI patients. One-way analyses of variance (parametric ANOVA and non-parametric Kruskal-Wallis test), followed by parametric (using *t*-test with pooled standard deviation) or non-parametric (using Wilcoxon rank sum test) *post-hoc* pairwise comparison tests with Bonferroni correction for multiplicity were applied to evaluate the differences in ONSD concerning age groups. A multivariable linear model was applied to verify whether age, sex and arterial blood pressure were independently related to ONSD in both healthy volunteers and TBI cohorts (following the linear model structure: *ONSD* ~ *age* + *sex* + *ABP)*. In the TBI cohort, we also verified whether intracranial pressure alongside with age, sex and arterial blood pressure were independently related to ONSD (following the linear model structure: *ONSD* ~ *age* + *sex* + *ABP* + *ICP)*. ABP and ICP were included as outcome measures to account for their modulating effect on global cerebral hemodynamics. The level of significance was set at 0.05.

## Results

The characteristics of the cohorts of healthy volunteers and TBI patients and the median (IQR) values of assessed variables are shown in Tables 1A–E. Table 2 presents the summaries of the multivariable linear models.

**Table 1A:** ONSD (mm) (median [IQR]) values in healthy volunteers and patients with traumatic brain injury (TBI) according to age groups and sex.

**Population**	**Volunteers**		**TBI patients**	
	4.2 (3.8–4.4)		4.5 (4.0–5.4)	
**Age Group**				
1	4.0 (3.7–4.2)		4.3 (3.9–5.3)	
2	4.2 (3.6–4.5)		4.8 (4.3–5.4)	
3	4.7 (4.2–4.8)		5 (3.8–5.4)	
**Sex**	**Males**	**Females**	**Males**	**Females**
	4.2 (3.9–4.6)	4.1 (3.6–4.2)	4.4 (3.9–5.0)	5.2 (4.3–6.2)
**Age Group**				
1	4.1 (3.8–4.4)	4.0 (3.6–4.2)	4.3 (3.9–5.1)	4.7 (4.2–5.6)
2	4.3 (4.2–4.8)	3.8 (3.6–4.2)	4.7 (4.3–5.0)	5.2 (4.2–6.7)
3	4.8 (4.5–4.9)	4.6 (4.2–4.27)	4.1 (3.8–5.0)	5.4 (5.4–5.5)

**Table 1B:** ABP (mm Hg) (median [IQR]) values in healthy volunteers and patients with traumatic brain injury (TBI) according to age groups and sex.

**Population**	**Volunteers**		**TBI patients**	
	84.8 (77.8–93.7)		86.7 (81.3–92.4)	
**Age Group**				
1	79.2 (72.8–87.8)		87.4 (82.0–92.4)	
2	87.7 (80.7–96.0)		86.1 (80.5–93.6)	
3	95 (89.3–104.0)		85.1 (80.2–89.3)	
**Sex**	**Males**	**Females**	**Males**	**Females**
	86.7 (78.2–94.7)	84.7 (76.3–92.8)	86.5 (80.4–92.1)	88.9 (82.8–93.5)
**Age Group**				
1	79.8 (75.7–88.2)	78.3 (70.8–85.0)	86.9 (81.8–91.7)	91.4 (84.1–95.5)
2	86.7 (80.3–97.0)	88.3 (81.7–94.0)	85.5 (80.0–93.6)	87.7 (82.4–92.6)
3	102.5 (92.5–109.6)	91.3 (86.5–99.8)	85.0 (77.6–89.2)	85.1 (82.8–89.4)

**Table 1C:** ICP (mm Hg) (median [IQR]) values in patients with traumatic brain injury (TBI) according to age groups and sex.

**Population**	**TBI patients**	
	10.69 (7.31–13.95)	
**Age Group**		
1	10.82 (8.04–13.53)	
2	10.51 (8.00–12.59)	
3	8.04 (6.50–12.15)	
**Sex**	**Males**	**Females**
	10.73 (7.09–13.42)	10.08 (8.04–13.26)
**Age Group**		
1	10.82 (7.86–13.48)	10.47 (9.36–13.99)
2	10.52 (7.09–12.47)	10.18 (9.11–12.59)
3	9.10 (6.43–11.60)	8.04 (7.04–13.08)

**Table 1D:** Age (years) (median [IQR]) values in healthy volunteers and patients with traumatic brain injury (TBI) according to age groups and sex.

**Population**	**Volunteers**		**TBI patients**	
	45.0 (32.2–57.2)		43.0 (28.0–61.0)	
**Age Group**				
1	31.0 (24.7–38.0)		29.5 (21.0–41.25)	
2	50.0 (46.0–55.0)		54.0 (51.7–61.0)	
3	72.0 (67.0–76.0)		72.0 (66.5–78.0)	
**Sex**	**Males**	**Females**	**Males**	**Females**
	44.5 (31.5–58.5)	45.0 (33.0–54.5)	43.0 (28.0–61.0)	48.0 (34.0–59.0)
**Age Group**				
1	31.0 (25.0–38.7)	31.0 (23.0–37.25)	29.5 (20.7–41.2)	31.0 (26.2–41.2)
2	52.0 (48.75–58.5)	48.0 (46.0–53.0)	54.0 (50.7–62.0)	54.0 (53.0–58.2)
3	71.0 (65.0–74.0)	72.0 (69.5–76.5)	73.0 (66.2–80.2)	68.0 (67.0–78.0)

**Table 1E:** Sample size of cohorts in healthy volunteers and patients with traumatic brain injury (TBI) according to age groups and sex.

**Population**	**Volunteers**		**TBI patients**	
	122		95	
**Age Group**				
1	60		52	
2	41		28	
3	21		15	
**Sex**	**Males**	**Females**	**Males**	**Females**
	60	62	70	25
**Age Group**				
1	30	30	40	12
2	20	21	20	8
3	10	11	10	5

**Table 2:** Summary of the multivariable linear models in healthy volunteers and patients with traumatic brain injury (TBI).

**Dependent variables**	**Parameter**	**Estimate**	**Std. Error**	***p*-value**	**95% Confidence Interval**	
					**Lower Bound**	**Upper Bound**
	**Healthy Volunteers**					
ONSD	*Intercept*	3.29	0.35	**<0.0001**	2.59	4.00
	*Age*	0.02	0.003	**<0.0001**	0.01	0.02
*R*^2^ = 0.30	*Sex*	−0.25	0.09	**0.005**	−0.43	−0.08
	*ABP*	0.002	0.005	0.55	−0.006	0.01
	**TBI Patients**					
ONSD	*Intercept*	0.95	1.55	0.54	−2.12	4.03
	*Age*	0.008	0.008	0.27	−0.007	0.02
*R*^2^ = 0.14	*Sex*	0.41	0.31	0.20	−0.21	1.03
	*ABP*	0.03	0.02	0.06	−0.001	0.07
	*ICP*	0.04	0.02	0.07	−0.002	0.08

*ONSD, optic nerve sheath diameter; ABP, mean arterial blood pressure, ICP, intracranial pressure*.

*Bold values indicate statistical significance (p < 0.05)*.

### Healthy Volunteers

**Figure 1:**
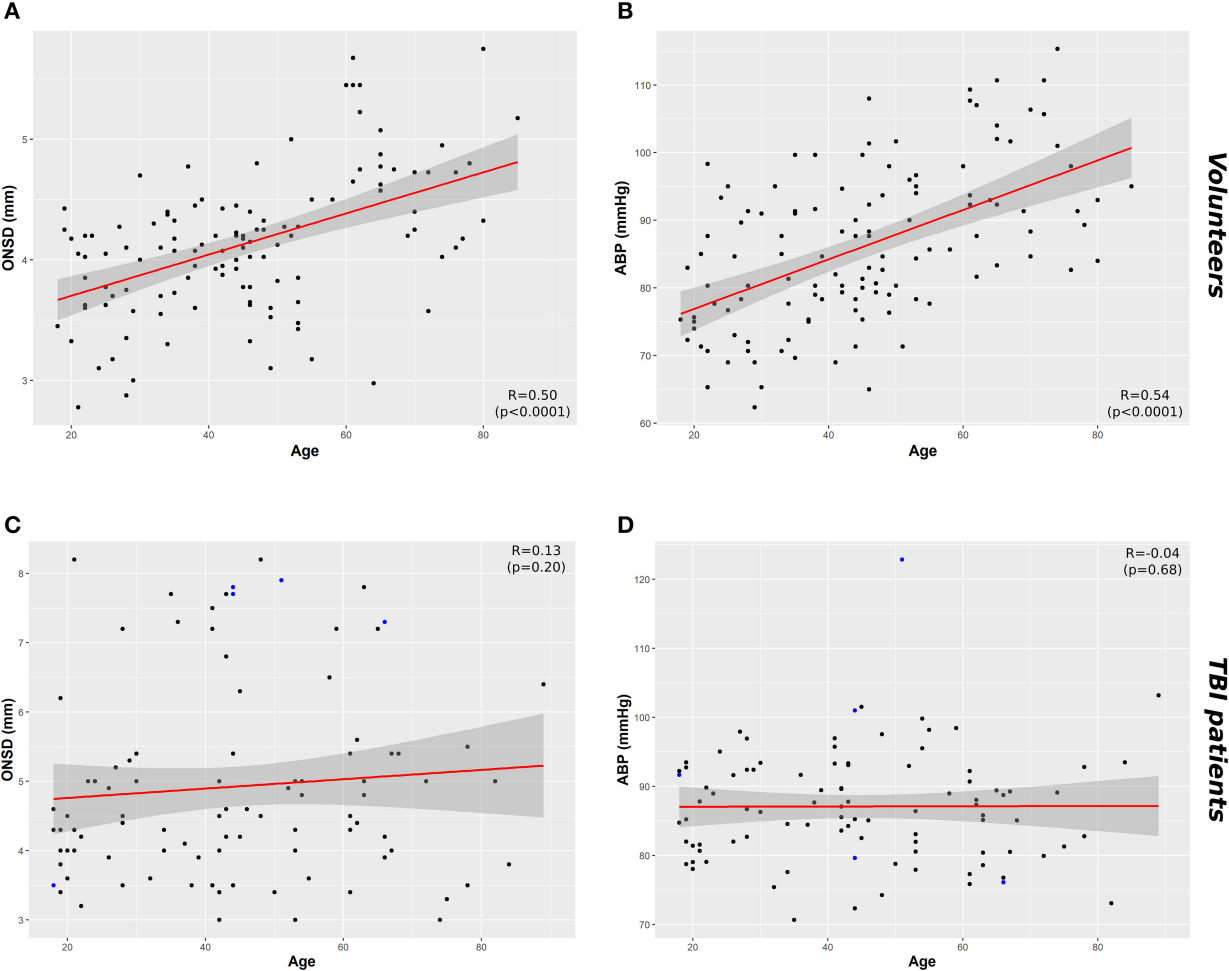
Scatterplots of ONSD (mm) and ABP (mm Hg) in healthy volunteers and TBI patients. **(A)** Correlation between ONSD and age in healthy volunteers (R = 0.50, *p* < 0.0001) and **(C)** in TBI population (R = 0.13 *p* = 0.20). **(B)** Correlation between age and ABP in healthy population (R = 0.54, *p* < 0.0001) and **(D)** TBI population (R = 0.04, *p* = 0.68). Data points highlighted in blue in **(C)** and **(D)** indicate the cases in which ICP > 20 mm Hg. Dark gray shaded areas on the plots represent 95% confidence intervals for the linear regressions; ABP, arterial blood pressure; ICP, intracranial pressure; ONSD, optic nerve sheath diameter; TBI, traumatic brain injury.

**Figure 2:**
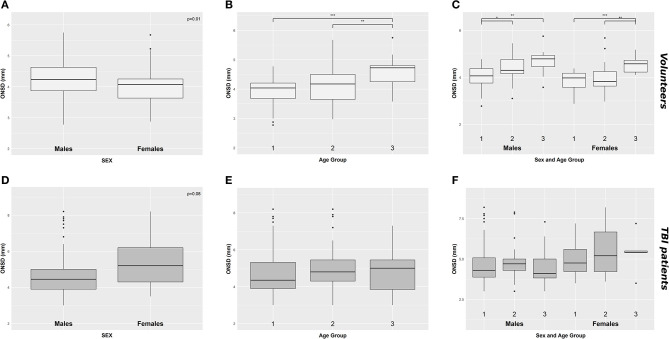
Boxplot demonstrating the differences of ONSD in healthy **(A–C)** and TBI population **(D–F)** according to sex **(A,D)**, age groups **(B,E)**, and considering both sex and age **(C,F)**. ONSD, optic nerve sheath diameter; *p < 0.05; **p < 0.01; ***p < 0.001; TBI, traumatic brain injury.

A significant positive correlation was found between ONSD and age (R = 0.50, *p* < 0.0001) (Figure 1A) and ABP and age (R = 0.54, *p* < 0.0001) (Figure 1B). ONSD was significantly different between females and males [4.1 (3.6–4.2) mm vs. 4.2 (3.9–4.6) mm (*p* = 0.01), respectively] (Figure 2A). Considering the entire population (without sex stratification), ONSD showed a statistically significant difference between age groups 1 and 3 [4.0 (3.7–4.2) mm vs. 4.7 (4.2–4.8) mm, *p* < 0.0001] and groups 2 and 3 [4.2 (3.6–4.5) mm vs. 4.7 (4.8–4.2) mm, *p* = 0.01] (Figure 2B) (Table 1A). Considering only males, ONSD was significantly different between age groups 1 vs. 2 [4.1 (3.8–4.4) mm vs. 4.3 (4.2–4.8) mm, *p* = 0.05] and 1 vs. 3 [4.1 (3.8–4.4) mm vs. 4.8 (4.5–4.9) mm, *p* = 0.004] (Figure 2C) (Table 1A). Considering only the female population, ONSD was significantly different between age groups 1 vs. 3 [4.0 (3.6–4.2) mm vs. 4.6 (4.2–4.7) mm, *p* < 0.001] and 2 vs. 3 [3.8 (3.6–4.2) mm vs. 4.6 (4.2–4.7) mm, *p* = 0.01] (Figure 2C) (Table 1A). ABP was significantly different between age groups 1 vs. 2 (*p* = 0.0002) and 1 vs. 3 (*p* < 0.0001) [79.17 (72.83–87.83) mm Hg, 87.67 (80.67–96.00) mm Hg and 95.00 (89.33–104.00) mm Hg, respectively for groups 1, 2, and 3] (Table 1B). ABP did not differ between males and females (*p* = 0.29) (Table 1B). The multivariable linear model analysis revealed that age and sex were independently related to ONSD (*p* < 0.0001 and *p* = 0.005, respectively) (Table 2).

### TBI Patients

We did not find a significant correlation between ONSD and age (R = 0.13, *p* = 0.20) (Figure 1C) or ABP and age (R = −0.04, *p* = 0.68) (Figure 1D). For illustrative purposes, the cases presenting ICP > 20 mm Hg are highlighted in blue in Figures 1C,D. No differences in ONSD were found among females and males of different age groups [5.2 (4.3–6.2) mm vs. 4.4 (3.9–5.0) mm (*p* = 0.08), respectively] (Figure 2D) (Table 1A). Moreover, ONSD was not significantly different among the age groups (Figures 2E,F) (Table 1A). ABP did not differ across the age spectrum or sexes (Table 1B). In this cohort, ICP was below the threshold for intracranial hypertension at patient admission (ICP > 20 mm Hg) (Table 1C), except for 5 cases (4 patients in the ICP range of 20–22 mm Hg and one with ICP > 70 mm Hg). The multivariable linear model analysis did not reveal any independently related variables to ONSD (Table 2). However, we observed borderline *p*-values for ABP and ICP (*p* = 0.06 and *p* = 0.07, respectively), which corroborates the positive and significant correlation between ONSD and these variables [R = 0.25 (*p* = 0.01) for ABP and R = 0.42 (*p* < 0.0001) for ICP; Figure S1].

### Comparison Between Healthy Volunteers and TBI Patients

A statistically significant difference in ONSD was found between the population of healthy volunteers and TBI patients [4.2 (3.8–4.4) vs. 4.5 (4.0–5.4) mm (*p* < 0.0001), respectively], with no ABP difference between the two cohorts.

**Figure 3:**
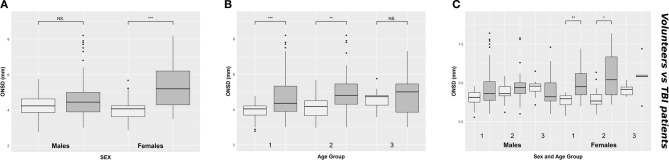
Boxplot comparing healthy (light gray) and TBI patients (dark gray) values of ONSD, according to sex **(A)**, age groups **(B)**, and considering both sex and age groups **(C)**. ONSD, optic nerve sheath diameter; NS. indicates *p* > 0.05; *p < 0.05; **p < 0.01; ***p < 0.001; TBI, traumatic brain injury.

ONSD was significantly different within females [4.1 (3.6–4.2) mm vs. 5.2 (4.3–6.2) mm (*p* < 0.0001), respectively for volunteers and patients] but not within males [4.2 (3.9–4.6) mm vs. 4.4 (3.9–5.0) mm (*p* = 0.12), respectively for volunteers and patients] (Figure 3A) (Table 1A). Considering age differences, ONSD was significantly higher in TBI patients of groups 1 (*p* < 0.0001) and 2 (*p* < 0.0001) (Figure 3B) (Table 1A). Considering sex and age differences, ONSD was only larger in female TBI patients in comparison to female volunteers of age groups 1 and 2 (*p* = 0.001 and *p* = 0.01, respectively) (Figure 3C) (Table 1A).

## Discussion

In the present study, we found that: (1) in healthy volunteers, ONSD was larger in males compared to females and increased with age; (2) in TBI patients, sex and age did not affect ONSD. Our findings suggest that different ONSD cut-off values considering sex and age are not relevant for the non-invasive assessment of increased ICP in TBI patients.

Several studies have previously attempted to investigate the normal values of ONSD in healthy individuals. Generally, they have suggested that ONSD remains relatively constant during the adult life (12–17), however, our results indicate that ONSD increases with age. This finding could be related to age-related physiological changes in the cerebrospinal fluid circulation, such as increases in CSF stroke and forward flow volumes in elderly individuals (18), possibly leading to an increased CSF volume in the subarachnoid space surrounding the optic nerve which would then produce an enlarged ONSD.

Previously, Kim et al. (19) studied a cohort of 585 healthy adults, finding a significant correlation of ONSD with sex, height and eyeball transverse diameter (ETD) in a simple linear regression analyses, but multiple linear regression analysis revealed only ETD to be independently associated with ONSD. However, this study had the limitation of having recruited only young adults (18–30 years), therefore not considering age differences across the cohort. Goeres et al. (17) studied a population of 120 healthy adults, finding that mean ONSD did not vary with age, weight or height but varied with sex wherein males also presented significantly larger ONSD than females. On the other hand, a study in 400 Nigerian healthy adults (20) showed no significant correlation of ONSD with age, sex, height, weight and measurement side (right and left eyes). In regard to the effect of sex in healthy volunteers, few studies have explored the nature of this relationship and it appears to be associated with variations in optic nerve fiber density between sexes (21).

Other studies have suggested that the optimal ONSD cut-off relating to increased ICP should be tailored to different ethnic groups. For instance, Wang et al. (15) studied ONSD in 230 Chinese adults, finding that the upper ONSD limit was lower than those in previous studies in Caucasian and African populations. They also found that underweight women had the smallest ONSD values, underlining differences due to sex, body mass index and ethnicity. Maude et al. (13) found a relatively higher and narrower range of ONSD (4.24–4.83 mm) in a mixed cohort of healthy Bangladeshi adult and children in contrast to the ranges previously reported in western healthy volunteers [for instance, 2.5–4.1 mm in 50 UK adults (22) and 2.1–4.3 mm in 102 UK children (23)]. In this scenario, the potential issue of ethnic differences in ONSD should be pondered in the interpretation of our results.

A novelty of our study is the assessment of differences concerning sex and age in TBI patients. The importance of describing such differences in this group of patients relies on ONSD being extensively reported as a predictor of increased ICP. In addition, understanding whether an appropriate threshold should be considered to account for potential confounds of age and sex is fundamental to the application of this technique for the non-invasive assessment of ICP. However, there is an ongoing debate about the optimal thresholds of intracranial hypertension itself to be used in clinical practice and this uncertainty would further affect the cut-off for pathological ONSD values. Most studies have investigated ONSD values considering the threshold for intracranial hypertension as ICP > 20 mm Hg (8, 10, 24–26), whereas few others used a higher threshold (ICP > 25 mm Hg) (27, 28). Moreover, there is another element of heterogeneity attributable to some studies wherein the reference value was ICP > 20 cm H_2_O or ICP > 25 cm H_2_O instead of mm Hg (9, 29, 30), as these values are not equivalent (20 mm Hg corresponds to 27.19 cm H_2_O, and 25 cm H_2_O corresponds to 18.39 mm Hg). A recent meta-analysis that evaluated the diagnostic accuracy of ultrasound measurement of ONSD for the assessment of intracranial hypertension found that ONSD cut-off values varied from 4.80 to 6.30 mm (10). Therefore, given the intrinsic variability this parameter might present, ONSD should be considered more as a trend assessed over time rather than “a single number.”

Furthermore, the conventionally accepted threshold for intracranial hypertension as ICP > 20 mm Hg (31) has been recently challenged due to discrepancies observed among different centers. For example, the DECRA trial randomized patients to decompressive craniectomy using an ICP threshold > 20 mm Hg even for a short duration (>15 min within 1 h-period) (32). In the RESCUE-ICP trial, patients underwent craniectomy when their ICP was sustained above 25 mm Hg for hours (1–12 h) (33). The latest American Brain Trauma Foundation guidelines for severe TBI proposed an ICP threshold of >22 mm Hg. This limit was derived from a single centre's (Cambridge, UK) retrospective study whose aim was not to determine a threshold for intracranial hypertension but to report an association between a single summary ICP value and 6 month outcomes after TBI (34). Moreover, Güiza et al. (35) showed that ICP is not only important as “a number” since the duration of intracranial hypertension has been associated with worse outcome, which supports the concept of “dose of ICP” (the magnitude and duration of ICP values above a pathologic threshold). In summary, there has not been a consensus about the optimal threshold for intracranial hypertension, and decisions regarding treatment and therapy escalation should be taken with a combination of ICP values, multimodal monitoring, clinical assessment and brain computed tomography findings (36).

In light of the above considerations, we demonstrated that different ONSD cut-off values considering age and sex do not appear to be relevant for the assessment of increased ICP in TBI patients. Similarly to the recommendations for determining a threshold for intracranial hypertension, ONSD should be assessed in combination with clinical findings as well as a trend over the course of patient management following the previously reported ONSD ranges associated with elevated ICP (10, 37).

Our study presents several limitations. Firstly, the data were collected by two operators in the two different centers. Although they were experienced and had equivalent level of training, we did not consider the interobserver variability as a potential confounder since the operators performed measurements in different individuals. Secondly, although reasonably expected in the TBI patient population given the heterogeneous incidence of this condition (38), the sample size of the TBI cohort was not homogeneous in terms of age and sex, and this could represent a source of bias. We also investigated whether age and sex alongside with ABP and ICP would be independently related to ONSD in a multivariable linear model analysis, based on the inference that TBI patients would present higher ONSD levels due to elevated ICP regardless of their age or sex. Nevertheless, none of these parameters were independently related to ONSD, which disregards ICP as a potential confounding factor. Moreover, in TBI patients, arterial blood pressure and intracranial pressure were actively controlled as part of the institutional management protocol, therefore the variability across individuals was small. In respect to age and sex not being independent predictors of ONSD in TBI patients, this could represent a type-I statistical error given the small sample size applied to a multivariable model adjusted for 4 variables (ICP, ABP, sex, and age). Finally, differences in ONSD values concerning ethnicity might be relevant in our comparison of healthy volunteers and TBI patients, and further studies with larger and more homogeneous recruitments are required to elucidate this potential confounding factor.

## Data Availability Statement

The raw data supporting the conclusions of this article will be made available by the authors, without undue reservation.

## Ethics Statement

The studies involving human participants were reviewed and approved by Research Ethics Boards of Genova, Italy (REC 031R8G2015) Research Ethics Boards at the University of Cambridge (REC 15/lo/1918). The patients/participants provided their written informed consent to participate in this study.

## Author Contributions

CR, DC, PH, and MC conceived the study design. DC and CR contributed to data acquisition, analysis, and/or interpretation. DC, CR, and KC drafted the manuscript. All authors critically reviewed the manuscript for important intellectual content and approved the manuscript for submission.

## Conflict of Interest

The authors declare that the research was conducted in the absence of any commercial or financial relationships that could be construed as a potential conflict of interest. The reviewer LL declared a past co-authorship with one of the authors PH to the handling editor.

## Supplementary Material

Figure S1 Scatterplot of the relationship between ONSD and ICP in TBI patients. ICP, intracranial pressure; ONSD, optic nerve sheath diameter; TBI, traumatic brain injury; R indicates the Spearman correlation coefficient for this relationship.
