# Supplementary figures and images for: Effects of Age and Sex on Optic Nerve Sheath Diameter in Healthy Volunteers and Patients With Traumatic Brain Injury
Source: Front Neurol. 2020 Aug 7;11:764. doi: 10.3389/fneur.2020.00764 (PMC7426488; doi:10.3389/fneur.2020.00764)

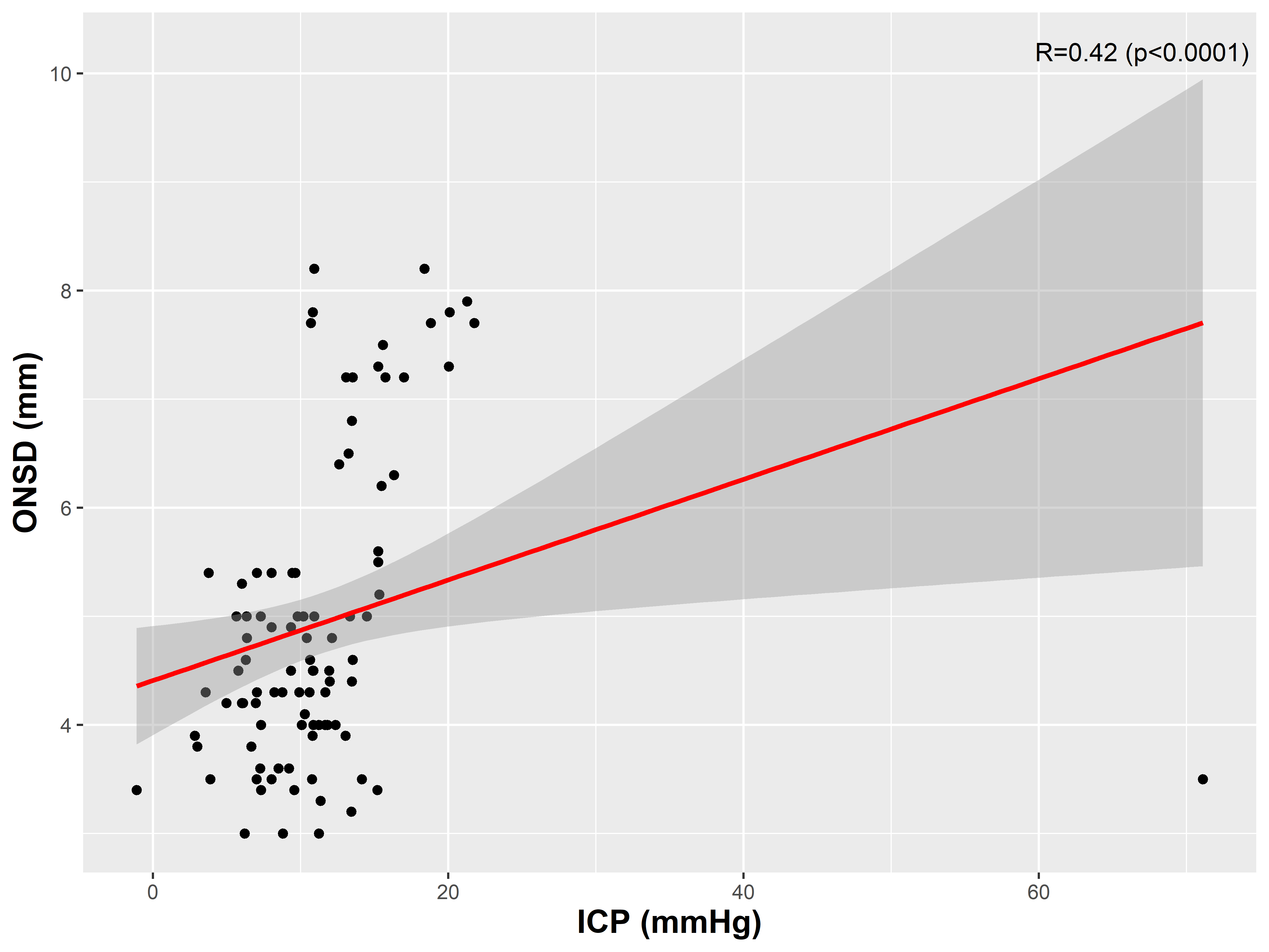

Supplement: Figure S1 — Scatterplot of the relationship between ONSD and ICP in TBI patients. ICP, intracranial pressure; ONSD, optic nerve sheath diameter; TBI, traumatic brain injury; R indicates the Spearman correlation coefficient for this relationship. [file Image_1.TIFF]
